# Evaluation of antibacterial efficacy of Chitosan, Chlorhexidine, Propolis and Sodium hypochlorite on *Enterococcus faecalis* biofilm : An *in vitro* study

**DOI:** 10.4317/jced.53777

**Published:** 2017-09-01

**Authors:** Natasha Jaiswal, Dakshita-Joy Sinha, Udai-Pratap Singh, Kanwardeep Singh, Urja-Ahuja Jandial, Shivika Goel

**Affiliations:** 1 PG student, Department of Conservative Dentistry and Endodontics, Kothiwal dental college and research centre, Moradabad, Uttar Pradesh-244001; 2 Associate professor, Department of Conservative Dentistry and Endodontics, Kothiwal Dental College and Research Centre, Moradabad, Uttar Pradesh-244001; 3 Professor & Head, Department of Conservative Dentistry and Endodontics, Kothiwal Dental College and Research Centre, Moradabad, Uttar Pradesh-244001

## Abstract

**Background:**

Long term successful root canal treatment requires effective debridement and disinfection of root canal system. Persistent periradicular lesions are usually associated with Enterococccus faecalis. Prompt research for natural alternatives for irrigation is mainly due to the constant increase in antibiotic resistant strains and side effects caused by synthetic drugs. Sodium hypochlorite; the gold standard for irrigation has many disadvantages. Therefore, the present study was aimed to explore newer irrigants probably be as more effective and at the same time would be less irritating to the tissues than NaOCl.

**Material and Methods:**

Ninety extracted human mandibular premolars were biomechanically prepared, vertically sectioned, placed in tissue culture wells exposing the root canal surface to *E. faecalis* to form a biofilm. At the end of 3rd week, all groups were irrigated with 3 ml of test solutions and control for 10 minutes. The samples were then scraped with a scalpel, inoculated on tryptone soy agar plates and incubated for 24 hours at 37ºC. The plates were then subjected to digital colony counter and evaluated for *E. faecalis*growth. The growth was statistically analysed by ANOVA & Post Hoc Tukey tests.

**Results:**

Chitosan + Chlorhexidine, NaOCl and Chlorhexidine showed no statistically significant difference, whereas all the other inter‑group differences were statistically significant (*P*<0.05).

**Conclusions:**

Chitosan + Chlorhexidine, Chlorhexidine and Propolis were found to be as efficacious as sodium hypochlorite. The use of natural alternatives as root canal irrigation solutions might prove to be advantageous considering several unfavorable properties of NaOCl.

** Key words:**Antibacterial efficacy, Chitosan, Enterococcus faecalis, Root canal irrigation.

## Introduction

Primary endodontic infections are caused by necrotic pulp tissue colonized by microorganisms (1). Success of endodontic treatment depends on complete debridement and disinfection of root canal space. This is not always achieved completely because microorganisms may be found in root canals, dentinal tubules, apical ramifications, cementum or areas of root resorption thereby limiting the access of root canal systems by instruments and irrigants (2).

Irrigants are multifunctional in endodontic treatment and are required to have antimicrobial effects, dissolve organic matter in the canal and flush out loose debris (3). *Enterococcus faecalis* is a persistent organism of root canal system and frequently isolated in the root canals with pulpal infection (4). It plays important role in the etiology of periradicular lesions after root canal treatment and seen in 22-77% root canal failure cases. *E. faecalis* possesses certain virulence factors including lytic enzymes, cytolysin, aggregation substance, pheromones and lipoteichoic acid. It has been shown to adhere to host cells, express proteins that allow it to compete with other bacterial cells and alter host responses. *E. faecalis* is able to suppress the action of lymphocytes, potentially contributing to endodontic failure (5).

The term biofilm was introduced to designate the thin layered condensations of microbes that may occur on various surface structures in nature. Free-floating bacteria existing in an aqueous environment, so-called planktonic microorganisms are a prerequisite for biofilm formation. Such films may thus become established on any organic or inorganic surface substrate where planktonic microorganisms prevail in a water-based solution (6). Achieving predictable success of root canal treatment requires effective debridement and disinfection of root canal system and biofilm. Therefore, a biofilm model is worked upon in this study making it more clinically relevant.

Sodium hypochlorite has been the gold standard for irrigation because of its ability to dissolve organic matter and high antimicrobial potential. However, there are certain major drawbacks associated with the use of sodium hypochlorite i.e irritant to periapical tissues, stains instruments, unpleasant taste, high toxicity, corrosion of instruments, inability to remove smear layer, burning of surrounding tissues and reduction in elastic modulus and flexural strength of dentin (7,8).

Propolis is also known as bee glue and bee propolis, is a brownish resinous substance collected by bees, mainly from plant (Apis mellifera L) around their hive, used to reinforce the combs and to keep the hive environment aseptic. It is a potent antioxidant, anti-inflammatory and anti microbial agent. In dentistry, propolis has been used for surgical wound repair, root canal irrigation, direct and indirect pulp capping reduction of dentin hypersensitivity in caries prevention against *Streptococcus mutans* and as a storage media for avulsed teeth. Ethanolic extract of propolis has proved to be an effective intracanal medicament in teeth infected with *E. faecalis* (9,10).

Chlorhexidine (CHX) is a broad spectrum antimicrobial agent that has substantive antimicrobial activity and relatively low toxic effects. However does not dissolve organic tissues (11). *In vitro* studies have shown CHX to exhibit sustained antimicrobial activity in the root canal for some time after being used as an endodontic irrigant. Therefore, CHX has been suggested as a root canal irrigant owing to its unique ability to bind to dentin, its effectiveness as an antimicrobial agent, and its substantivity in the root canal system (12).

Chitosan is a natural polysaccharide comprising of copolymers of glucosamine and N-acetylglucosamine. Partial deacetylation of chitin results in production of chitosan. It is biocompatible, biodegradable, bioadhesive and there is no reported toxicity. Besides it is a good antimicrobial agent. Its low production costs has increased its utility for various applications in the areas of medicine and pharmaceuticals. In dentistry it has been used as a barrier membrane for periodontal therapy and as oral mucosal delivery agent for chlorhexidine. In addition it has high chelating capacity for different metal ions in acidic conditions. In a study conducted by Silva *et al.* (13) chitosan has effectively removed smear layer from the root canals after instrumentation. In endodontics its role as antibacterial and antifungal agent has not been subjected to adequate scrutiny. The possibility for its use as an irrigant in root canal treatment is yet to be investigated (14). 1% acetic acid was be used as one of the groups in the study as to find out whether the antimicrobial activity of chitosan is influenced by its addition.

Literature has shown that the antimicrobial efficacy of chlorhexidine increases when combined with chitosan for intracanal medication. But these studies evaluated the gel forms and this combination has not been used till date for root canal irrigation on *in vitro* tooth models or *in vivo* (13).

As there are inherent drawbacks of using conventional irrigants, therefore the purpose of this study was to evaluate other alternatives for root canal irrigation with high antimicrobial activity or atleast similar to that of conventional irrigants with low toxicity.

## Material and Methods

*E. faecalis* culture preparation

A pure culture of *Enterococcus faecalis* (ATCC 29212) [Himedia, Mumbai] was inoculated on Mueller-Hinton agar plates [Himdia, Mumbai] incubated at 37°C overnight and was adjusted to an optical density of one MacFarland on optical densitometer (Densicheck plus) with sterile brain heart infusion broth (Himedia). 

Test solutions preparation

5% sodium hypochlorite, 2% chlorhexidine, 1% acetic acid, Propolis, 0.2% Chitosan ,0.2% Chitosan + 2% Chlorhexidine, 1% chitosan + 1% chlorhexidine and 2% chitosan + 2% chlorhexidine groups were taken for the study. Saline was used as the negative control.

Propolis [HiTech natural products, New Delhi, India] was prepared by diluting a 33% commercially available alcoholic extract using warm saline in a ratio of 2:1, to form an 11% alcoholic extract.

For preparation of 0.2% chitosan solution (Thahira chemicals, Kerela, India), 0.2gm of chitosan was diluted in 100ml of 1% acetic acid.

For preparation of 1% chitosan solution, 1gm chitosan was diluted in 100ml of 1% acetic acid.

For preparation of 2% chitosan solution, 2gm chitosan was diluted in 100 ml of 1% acetic acid.

Chlorhexidine solution was prepared in 3 concentrations (0.2%, 1.0%, 2.0%) from 100% chlorhexidine provided by the manufacturer (Basic pharma, Gujarat, India).

5% sodium hypochlorite was used as provided by the manufacturer (Dentpro).

Tooth samples preparation

Ninety single rooted type I Vertucci’s classification human mandibular premolar teeth with fully formed apices were taken for the study. The specimens were cleaned of superficial debris, calculus, tissue tags and stored in normal saline. The specimens were sectioned below the cementoenamel junction with a diamond disk to obtain a standardized tooth length of 8 mm.

The root canals were then instrumented using the crown down technique and rotary instruments to an apical size of ProTaper F3. A total volume of 2 ml of 5% sodium hypochlorite (Dentpro) was used between each instrument during the cleaning and shaping procedure. All teeth were then vertically sectioned along the mid-sagittal plane into two halves. The concave tooth surface was minimally grounded to achieve flat surface to enable placement in tissue culture wells exposing the root canal surface to *Enterococcus faecalis* to form a biofilm.

Grouping and assessment protocol

The samples were then divided into nine experimental groups with 20 samples (after vertical sectioning) each and irrigated with 3 ml of each irrigant for 10 minutes.

• Group 1 - 5% Sodium hypochlorite (n=20; after vertical sectioning)

• Group 2 - 2% Chlorhexidine (n=20)

• Group 3 - 1% Acetic acid (n=20)

• Group 4 - Propolis (n=20)

• Group 5 - 0.2% Chitosan (n=20)

• Group 6 - 0.2%Chitosan+2%Chlorhexidine (n=20)

• Group 7 - 1% Chitosan+1%Chlorhexidine (n=20)

• Group 8 - 2%Chitosan+2%Chlorhexidine (n=20)

• Group 9 - Saline (negative control) (n=20)

The samples were then sterilized by ultraviolet radiation with a dosage of 300 kJ/cm2 in a biosafety cabinet for 10 minutes and placed in the wells of tissue culture plates.

The bacterium were then inoculated in 1ml of tryptone soy agar (Himedia) broth in 180 tissue culture wells and the turbidity was adjusted to 1 on the densitometer with sterile BHI broth taken as baseline. The sectioned tooth specimens were then placed in the tissue culture wells and inoculated at 37°C for 6 weeks.

At the end of 6th week of inoculation, all specimens were placed in sterile petridishes and the test irrigation solutions was delivered onto them using a micropipette.

**Figure 1:**
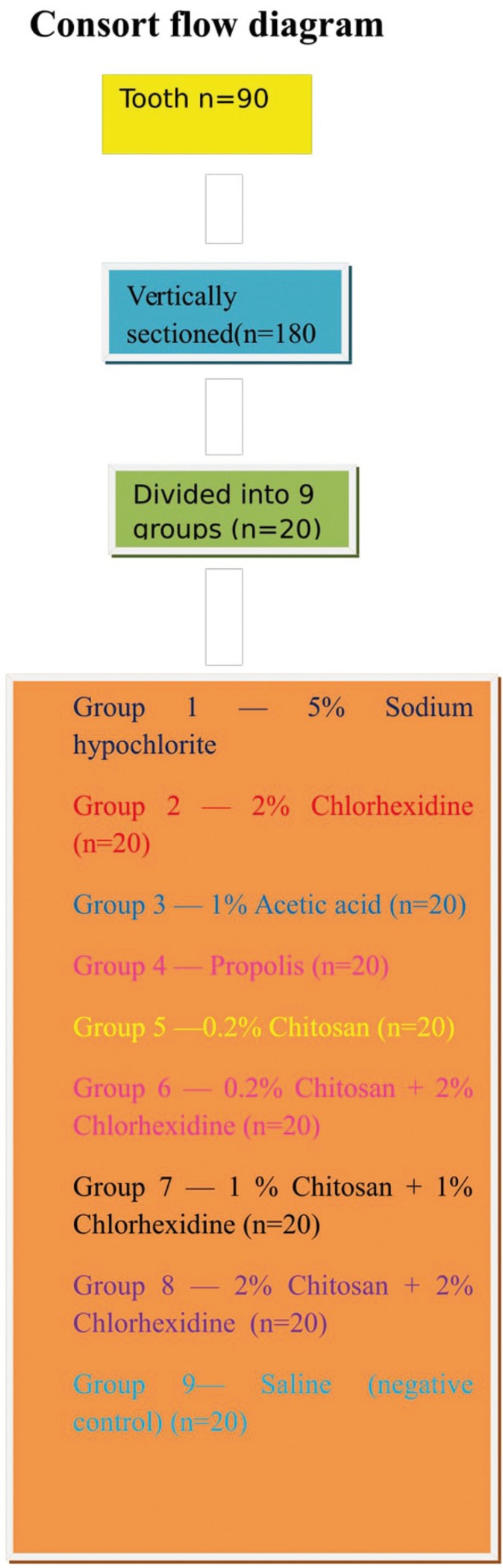
Flow consort diagram.

Then, the biofilm on root canal surface was taken with a sterile scalpel and inoculated on tryptone soy agar plates and incubated for 24 hours at 37°C. The plates were then analysed for colony forming units (108 cfu/ml) by a digital colony counter (Fig. 1). The data collected were subjected to the statistical analysis by using one way Anova analysis ( Table 1, Table 2) & intergroup comparison with post hoc tukey tests ( Table 3,  Table 3 continue,  Table 3 continue-1). Values of *p* < 0.05 were considered statistically significant.

**Table 1:**
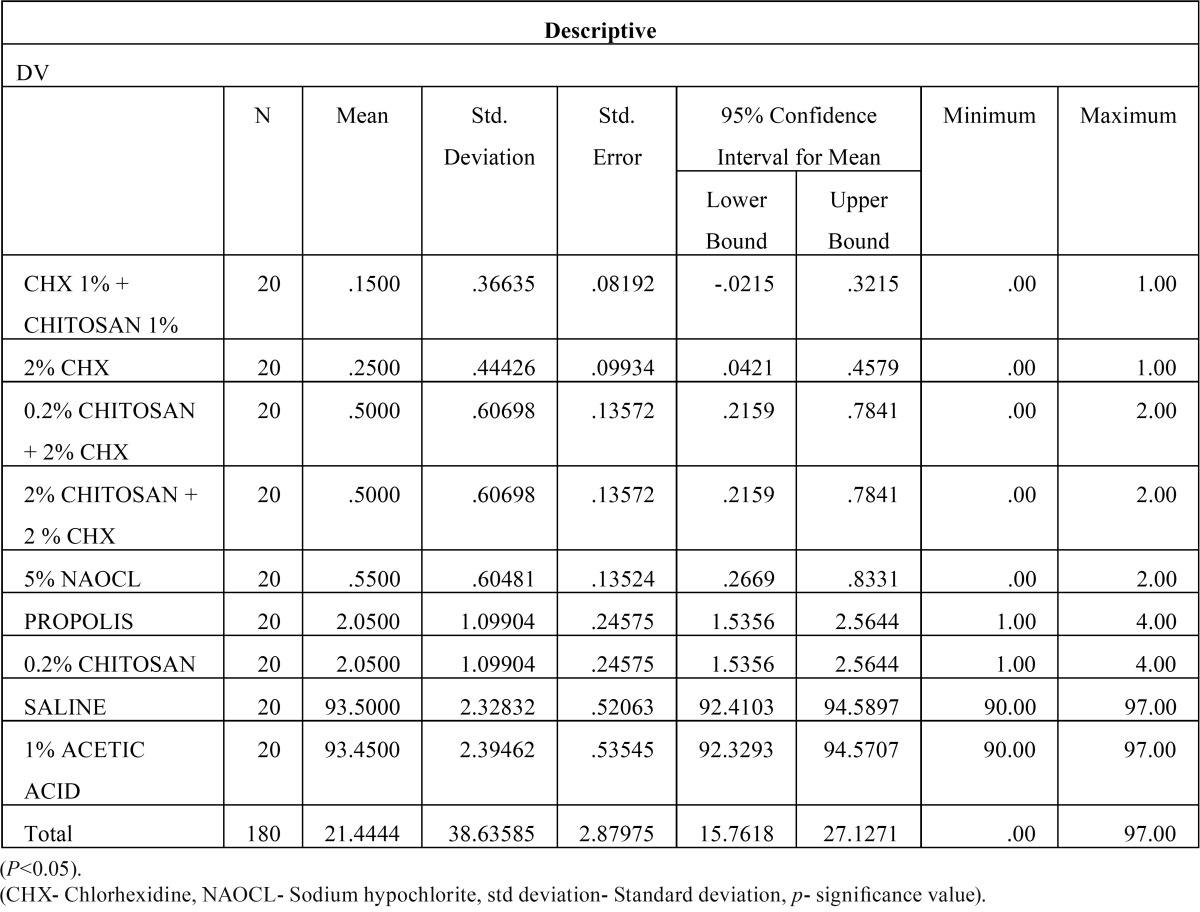
The mean and standard deviations obtained for all groups.

**Table 2:**
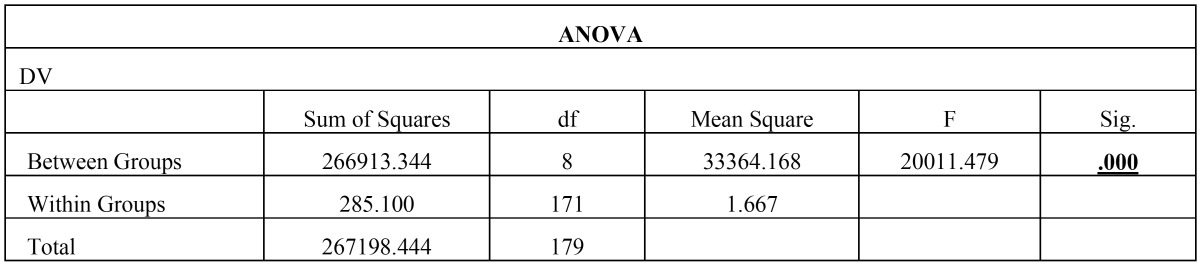
Result from ANOVA.

**Table 3:**
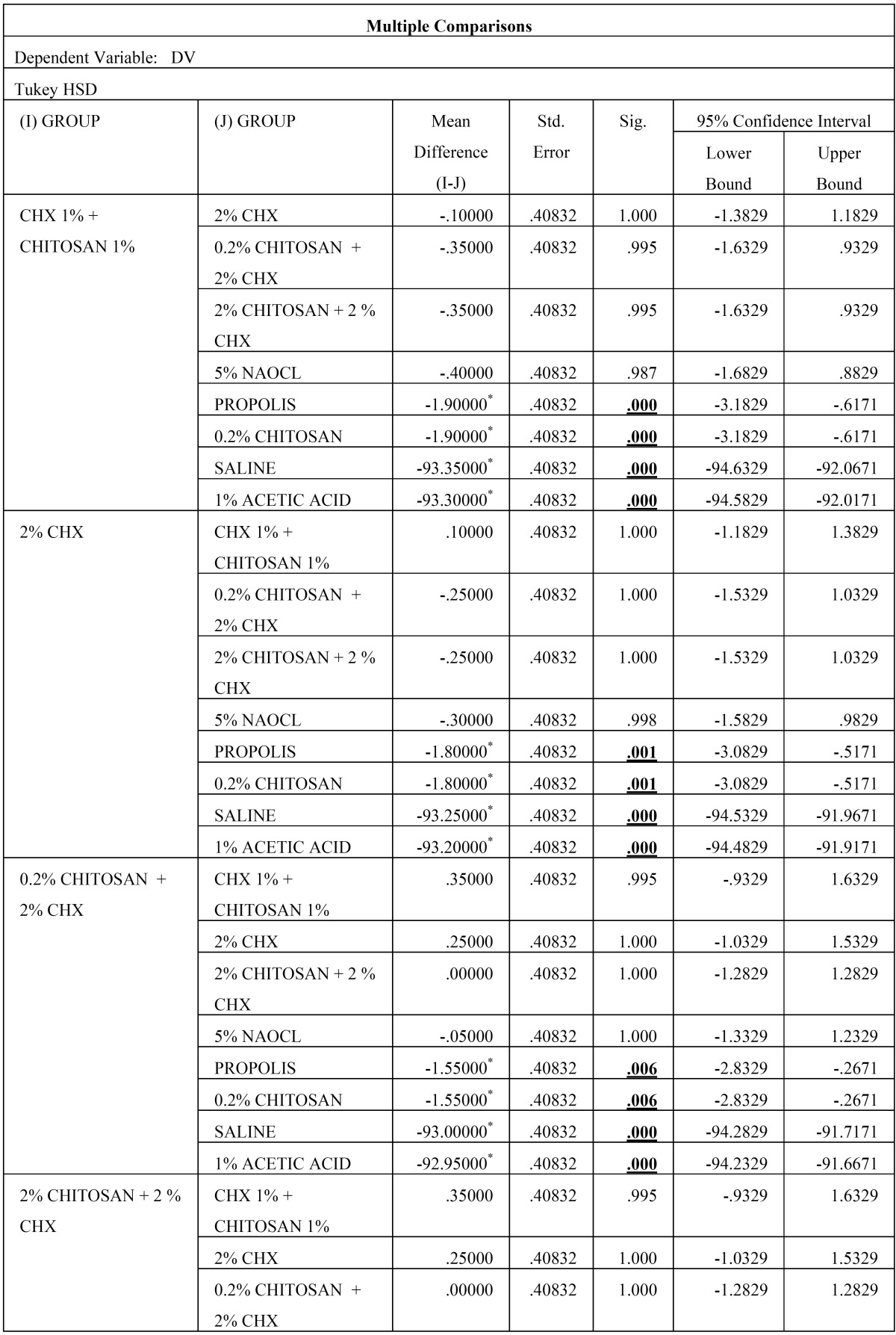
Results obtained from Post Hoc Analysis using Tukey’s t Test.

**Table 3 continue:**
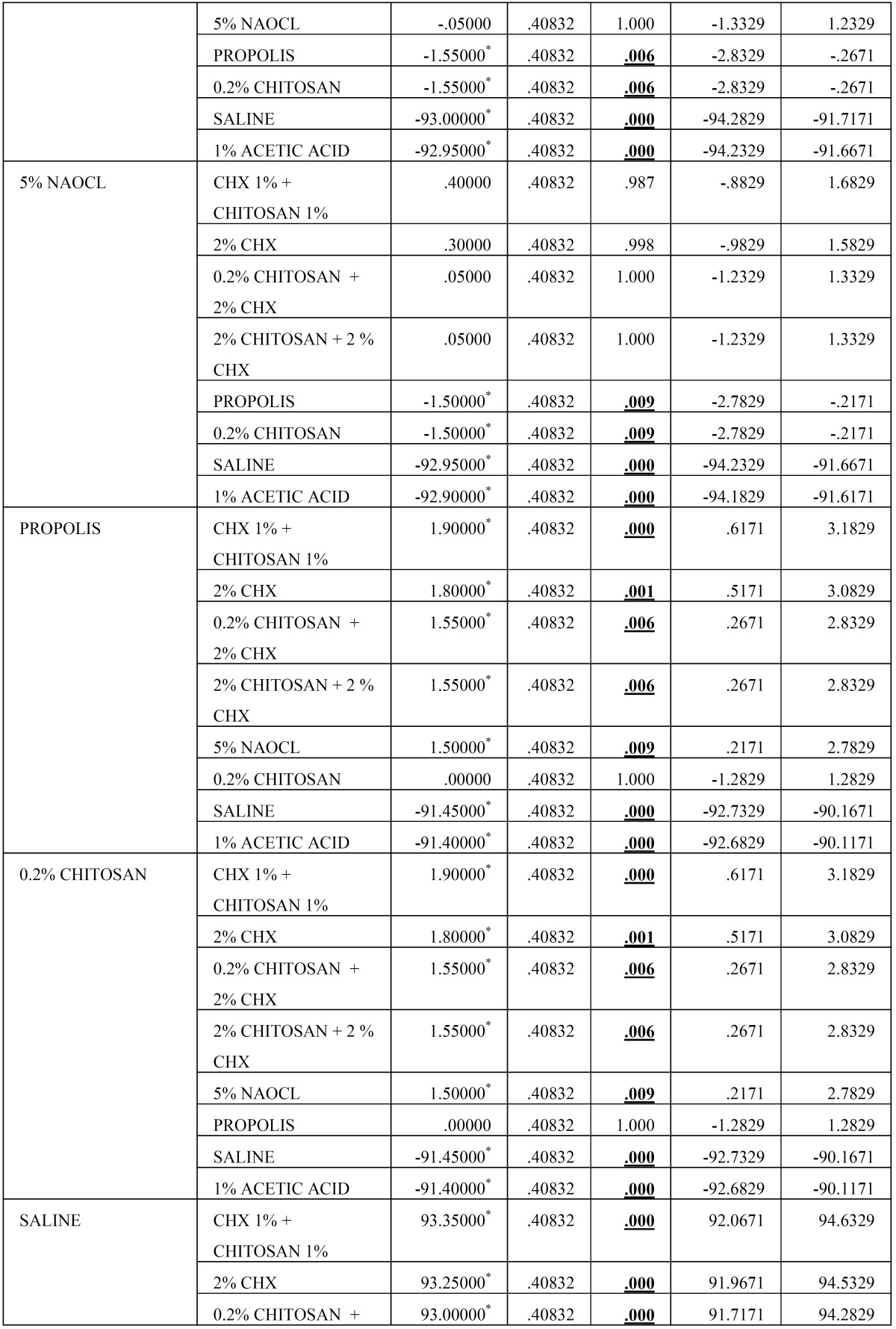
Results obtained from Post Hoc Analysis using Tukey’s t Test.

**Table 3 continue-1 continue:**
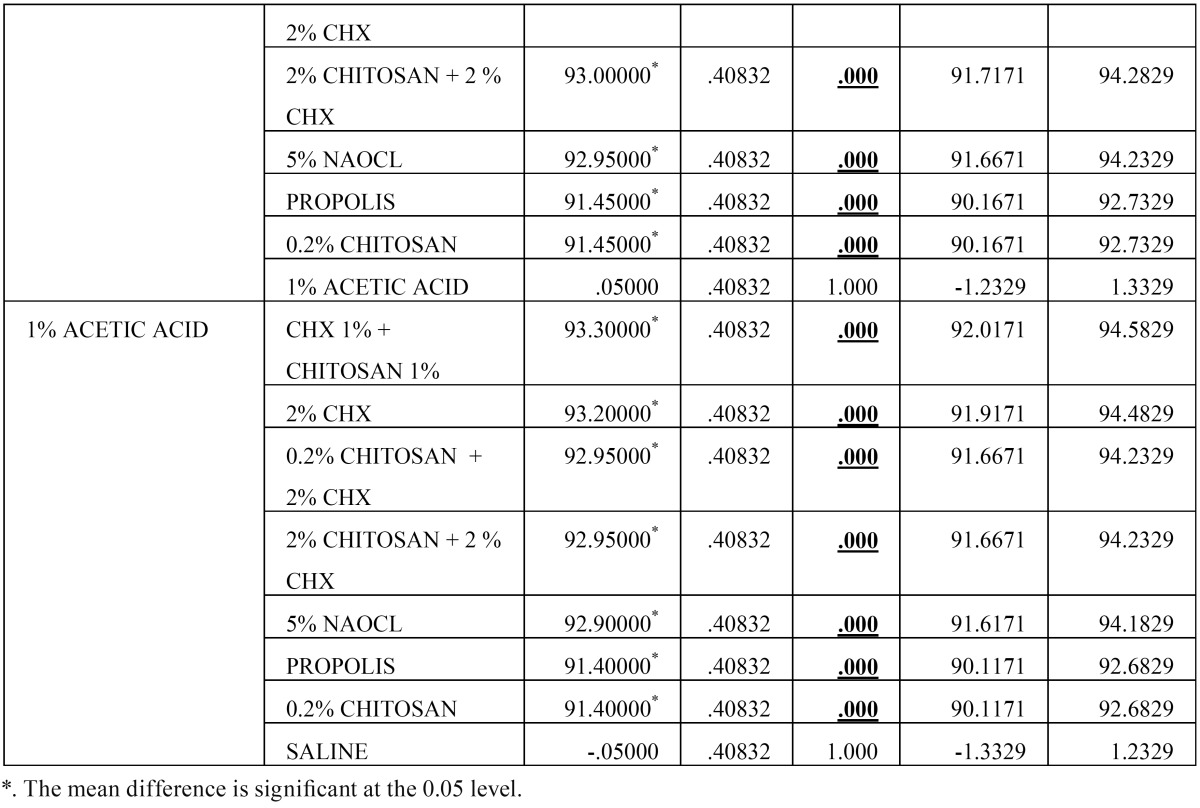
Results obtained from Post Hoc Analysis using Tukey’s t Test.

## Results

**Figure 2:**
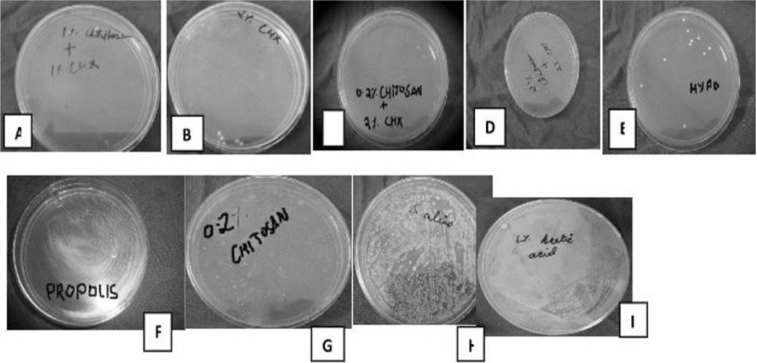
Growth of E.faecalis after irrigation with respective test solutionsi.e A) CHX 1% + Chitosan 1%, B) 2% CHXC) 0.2% Chitosan + 2% CHXD) 2% Chitosan + 2 % CHX, E) 5% NaOCl, F) Propolis, G) 0.2% Chitosan, H) Saline, I) 1% Acetic Acid.

From  Table 2 it is evident that the ANOVA is significant as *p* =0.00 < 0.05, and thus there is significant difference between the means of the different groups. The Post Hoc Analysis using Tukey’s t Test ( Table 3 and Fig. 2) evaluation showed that there is significant difference between the different groups of irrigants used against *E. faecalis*, however we can condense the above results in the following order 

(CHX 1% + CHITOSAN 1%) = (2% CHX) = (0.2% CHITOSAN + 2% CHX) = (2% CHITOSAN + 2 % CHX) = (5% NAOCL) < (PROPOLIS) = (0.2% CHITOSAN) < (SALINE) = (1% ACETIC ACID)

## Discussion

*Enterococcus faecalis* is the most common Enterococcus species isolated from root filled teeth with chronic apical periodontitis (15). Bacteria-induced dissolution of the dentin surface and the ability of *E. faecalis* to form calcified biofilm on root canal dentin may be a factor that contributes to their persistence after endodontic treatment (16). Hence, *E. faecalis* biofilm was formed on a tooth substrate in this study in accordance with the methodology done by Kishen *et al.* (17).

Clinical and laboratory studies have not demonstrated any significant difference in antibacterial effect between NaOCl concentrations ranging from 0.5% to5% (18) in the root canal (canal wall samples). Giardino *et al.* (19) demonstrated that 5.25% NaOCl eliminated *E. faecalis* biofilm in 30 seconds. Dunavant *et al.* (20) have shown that only NaOCl is able to kill the whole bacteria population organized in a biofilm. Though sodium hypochlorite has been found to be most potent endodontic irrigant but it has certain disadvantages and so to overcome them other alternatives are being incorporated. In our study NaOCl showed antimicrobial efficacy almost similar to combinations of 2% chlorhexidine and 2% chitosan.

Chlorhexidine digluconate (CHX) has been suggested as a root canal irrigant owing to its unique ability to bind to dentin, its effectiveness as an antibacerial agent against *E. faecalis* and its substantivity in the root canal system (21). In our study different concentrations of chlorhexidine has been used in combination with chitosan i.e 1% chitosan +1% chlorhexidine and 2% chlorhexidine alone. In both the cases the antimicrobial efficacy was achieved and was highest among all the groups.

Propolis is composed of 50% resin and vegetable balsam, 30% wax, 10% essential and aromatic oils, 5% pollen and 5% various other substances, including organic debris depending on the place and time of collection. Some components present in propolis extract, like flavonoids (quercetin, galangin, pinocembrin) and caffeic acid, benzoic acid, cinnamic acid, probably act on the microbial membrane or cell wall site, causing functional and structural damages (22). Kujumgiev *et al.* (23) reported the antimicrobial action of propolis to be due to flavonoids and esters of phenolic acids. In a study conducted by Al-Qathami and Al-Madi, the anti microbial efficacy of propolis, sodium hypochlorite and saline as endodontic irrigants was compared and it was found that propolis showed anti-microbial activity similar to that of sodium hypochlorite (24). In our study propolis showed antimicrobial efficacy which was similar to 0.2% chitosan alone.

Mechanism of action of chitosan is thought to be that cationically charged amino group may combine with anionic components such as N-acetyl muramic acid, sialic acid, and neuramic acid on the cell surface and suppresses growth of bacteria by impairing the exchanges with medium, chelating transition metal ions, and inhibiting enzymes. Therefore, chitosan has been added to chlorhexidine in an attempt to test the potential additive or synergistic effect on the viability of *E. faecalis*. The possible reason for the antimicrobial action of chitosan might be due to the mechanism of action of chitosan that possesses the positively charged NH3 + groups of glucosamine that interacts with negatively charged surface components of bacteria, resulting in extensive cell surface attraction, leakage of intracellular substances, and causing damage to vital bacterial activities (25). In a study conducted by Shaymaa *et al.*, Ca(OH)2 combined with chitosan solutions were more effective in inhibiting the growth of *E. faecalis* when compared with Ca(OH)2 mixed with saline (26). Ballal *et al.*, reported that 2% chlorhexidine (CHX) gel combined with chitosan has shown highest antimicrobial effect against *C. albicans* and *E. faecalis* when compared with CHX gel or 2% chitosan alone (14). In our study 1% chitosan was combined with 1% chlorhexine exhibited higher antimicrobial efficacy followed by other combinations such as 0.2% chitosan + 2% chlorhexidine and 2% chitosan and 2% chlorhexidine. Some authors believe that chitosan may have demineralizing effect but it has been also used as intracanal dressings which are given for 5-7 days and showed good antimicrobial effect. So, in our study for the first time it has been used as root canal irrigating solution which showed good antimicrobial effect.

In our study 1% acetic acid had similar results to those of the control group which is in support of the study conducted by Silva *et al.* in which they demonstrated that the chelation effect of Chitosan is due to its own properties rather than because of 1% acetic acid in which it is prepared (13) . The 0.2% chitosan solution, even in such a low concentration, was able to disinfect the root canal. It is important to emphasize that the antibacterial efficacy attributed to 0.2% chitosan were higher than those given to 1% acetic acid. Such information is important because the chitosan solution used in the present study was prepared using 1% acetic acid. Therefore, it is apparent that the antibacterial efficacy is attributed to the properties of chitosan and not of 1% acetic acid, which had a similar antimicrobial activity that of the control group. Saline was taken as negative control which as expected has least antimicrobial activity.

## Conclusions

Under the limitations of this study, it can be concluded • Combination of chitosan + chlorhexidine can be used as root canal irrigating solution among which 1% chitosan+ 1% chlorhexidine combination was shown to have better antimicrobial efficacy.

• Chlorhexidine is equally efficacious as combination of 1% chitosan + 1% chlorhexidine against *E. faecalis* biofilm.

• NaOCl performed equally well as that of 2% chitosan + 2% chlorhexidine.

• Propolis also exhibited significant antimicrobial activity.

Thus, from the results of the study, it can be suggested that all these three combinations of irrigating solutions i.e 1% chitosan+1% chlorhexidine, 0.2 chitosan+2% chlorhexidine and 2% chitosan+ 2% chlorhexidine could be used as an alternative to NaOCl for endodontic infections although, further *in vivo* long term studies are warranted.
